# Supplementary material for: New wave behaviors of the Fokas-Lenells model using three integration techniques
Source: PLoS One. 2023 Sep 11;18(9):e0291071. doi: 10.1371/journal.pone.0291071 (PMC10495015; doi:10.1371/journal.pone.0291071)
Supplement: Attachment — Submitted filename: PONE-D-23-23981review.pdf [file pone.0291071.s001.pdf]

Editor, PLOS ONE

Re: PONE-D-23-23981

Title: New wave behaviors of the Fokas-Lenells model using three integration techniques

Authors: M.S. Ullah, H.R. Roshid and M.Z. Ali

The paper presents the generation of wave solutions of the Fokas-Lenells equation.

Technically, the results are sound.

However, the solution method is based on several assumptions, for which no physical justification is provided. Here are a few.

1. The choice of Eq. (3.1) means that the authors are looking for a specific family of solutions. Are they of any physical significance? Why this choice?

2. The choice made in the line preceding Eq. (3.5) means that in the family of solutions discussed by the authors, is further reduced, with no discussion of the physical significance, or justification.

3. The choice of Eqs. (4.1) - (4.2) is not explained or justified, neither in the present paper, nor in Ref. [21].

4.The same criticism applies to the choice of Eqs. (5.1) - (5.2) and Ref. [22] and to Eqs. (6.1) - (6.2).

Even if my reservations are satisfactorily answered, my inclination is to recommend submission of the paper to a more specialized/technical journal, rather than PLOS-ONE, which aims at a far more general readership.
